# Digital Phenotyping of Parkinson’s Disease via Natural Language Processing

**DOI:** 10.21203/rs.3.rs-6017580/v1

**Published:** 2025-02-18

**Authors:** Simona Aresta, Petronilla Battista, Cinzia Palmirotta, Serena Tagliente, Gianvito Lagravinese, Paola Santacesaria, Allegra Benzini, Davide Mongelli, Brigida Minafra, Christian Lunetta, Adolfo M. García, Christian Salvatore

**Affiliations:** Istituti Clinici Scientifici Maugeri IRCCS, Laboratory of Neuropsychology, Institute of Bari; Istituti Clinici Scientifici Maugeri IRCCS, Laboratory of Neuropsychology, Institute of Bari; Istituti Clinici Scientifici Maugeri IRCCS, Laboratory of Neuropsychology, Institute of Bari; Istituti Clinici Scientifici Maugeri IRCCS, Laboratory of Neuropsychology, Institute of Bari; Istituti Clinici Scientifici Maugeri IRCCS, Laboratory of Neuropsychology, Institute of Bari; Istituti Clinici Scientifici Maugeri IRCCS, Laboratory of Neuropsychology, Institute of Bari; Istituti Clinici Scientifici Maugeri IRCCS, Laboratory of Neuropsychology, Institute of Bari; Istituti Clinici Scientifici Maugeri IRCCS, Laboratory of Neuropsychology, Institute of Bari; Istituti Clinici Scientifici Maugeri IRCCS, Laboratory of Neuropsychology, Institute of Bari; Istituti Clinici Scientifici Maugeri IRCCS, Department of Neurological Rehabilitation, Institute of Milan; Cognitive Neuroscience Center, Universidad de San Andrés; Department of Science, Technology and Society, University School for Advanced Studies IUSS Pavia

**Keywords:** Parkinson’s disease, mild cognitive impairment, multivariate language analysis, NLP, connected speech, linguistic biomarkers, machine learning

## Abstract

Frontostriatal degeneration in Parkinson’s disease (PD) is associated with language deficits, which can be identified using natural language processing, a remarkable tool for digital phenotyping. Current evidence is limited in linguistic coverage and mostly blind to the disorder’s cognitive phenotypes. We validated an AI-driven approach to capture digital language markers of PD with and without mild cognitive impairment (PD-MCI, PD-nMCI) relative to healthy controls (HCs). Analyzing the connected speech samples of participants, we extracted linguistic features with CLAN software. Classification was performed using Support Vector Machine and Recursive Feature Elimination. Discrimination between PD and HCs reached an AUC of 77%, with even better results for subgroup analyses (AUC 85% PD-nMCI vs. HCs; 83% PD-MCI vs. HCs; 75% PD-nMCI vs. PD-MCI). Key linguistic features included retracing ratio, action verb ratio, utterance error ratio, and verbless-utterance ratio, highlighting the foundational capabilities of linguistic digital markers for early diagnosis and phenotyping of PD.

## Introduction

Parkinson’s disease (PD) is a progressive neurological disorder resulting from the degeneration of dopamine-producing neurons in the nigrostriatal pathway due to extensive cell loss in the basal ganglia areas^1,2^. In addition to motor impairments, PD involves several cognitive deficits^3^, including linguistic alterations which can occur irrespective of dysarthria or other motor symptoms^4–6^. Indeed, multiple works argue for linguistic assessments as a key approach to capture early markers of the disorder^7–10^.

Linguistic deficits span across different domains in PD. At the phonological level (how speech sounds, i.e., phonemes, are combined to form words), patients may include phonological fragments, repetitions, revisions, and prolongation in a manner that interrupts the normal rhythm and flow of speech^11^. At the level of lexico-semantics (linking word forms with conceptual information), individuals with PD present systematic difficulties with action verbs (i.e., words that describe physical movement), as these hinge on the integrity of affected motor networks^12^. At the morpho-syntactic level (hierarchically organizing morphemes and words), compared to healthy controls (HCs), individuals with PD may exhibit difficulties in verb inflection^13^ or verb generation^14,15^, in word derivation for specific lexical classes^16^, patients may also show simpler sentence structures or a greater unpredictability in choosing grammatical alternative^7,8,17–19^. At the level of pragmatic and discourse organization patients show decreases in informativeness, correction of output errors, and pragmatic adequacy^20^.

Moreover, significant differences in the linguistic profile of PD have been found depending on the presence of cognitive impairment. Indeed patients without mild cognitive impairment (PD-nMCI) showed selective deficit in action verbs with high motion content compared to noun processing, conversely, individuals with mild cognitive impairment (PD-MCI) have been reported to be impaired across several linguistic domains, including lexico-semantic deficits with greater difficulties for action verb than noun processing^7,21^. It is still not clear whether those alterations are proportional to the degree of motor impairment or the patient’s cognitive status, as studies have yielded mixed findings on this issue^6,7,17,21–24^. Understanding the relationship between linguistic features and cognitive/motor severity in PD could provide insights into tracking disease progression.

Recently, digital linguistic markers, i.e., measurable features that can be computationally extracted and analyzed from connected/spontaneous speech, are emerging as a novel approach in PD^6,7,17,21–23^. Conected/spontaneous speech task allows examining language production beyond the single-word level and the digital linguistic features subsequently extracted circumvent the limitations of standard linguistic analysis, which typically is manually-based and therefore cannot have the same accuracy in calculating and processing large amounts of psycholinguistic features, which can be time-consuming if manually performed. Eyigoz et al. (2020) extended findings on digital linguistic features in PD by demonstrating that morphological errors can be automatically extracted from spontaneous speech to classify patients and have trans-linguistic validity, being distinctly affected across different languages such as Spanish, Czech, and German^22^. However, the evidence about the usefulness of assessing digital language markers in connected/spontaneous speech of PD is still scant and based only on a few languages, such as Spanish and English^25^, casting doubt on its usefulness for other languages. Moreover, most studies focus solely on circumscribed features, one at a time, failing to exploit their aggregate discriminatory potential and to reveal which of them best distinguish between PD-MCI and PD-nMCI while capturing core symptom severity across patients. To date, most studies using a digital framework focus mainly on the comparison between PD vs. HCs^22,26–31^, only the study of Garcia et al. 2022 discriminates between PD-nMCI and PD-MCI, yet using circumscribed features belonging to a specific linguistic domain (e.g, lexico-semantic). The use of multidomain features could help in revealing which of them best distinguishes between different cognitive phenotypes while capturing core symptom severity across patients. This approach can help in revealing which feature belonging to a specific linguistic domain has a greater impact in defining the linguistic impairment in the disease and can yield a better characterization of the deficits that patients with and without MCI can differentially show. Further, while linguistic deficits have been identified in PD, only a few studies have relied on automated tools (i.e., machine learning classifiers) which offer a powerful framework to analyze ecological tasks and identify hidden patterns in data in a cost- and time-efficient manner^32^.

**Figure 1:**
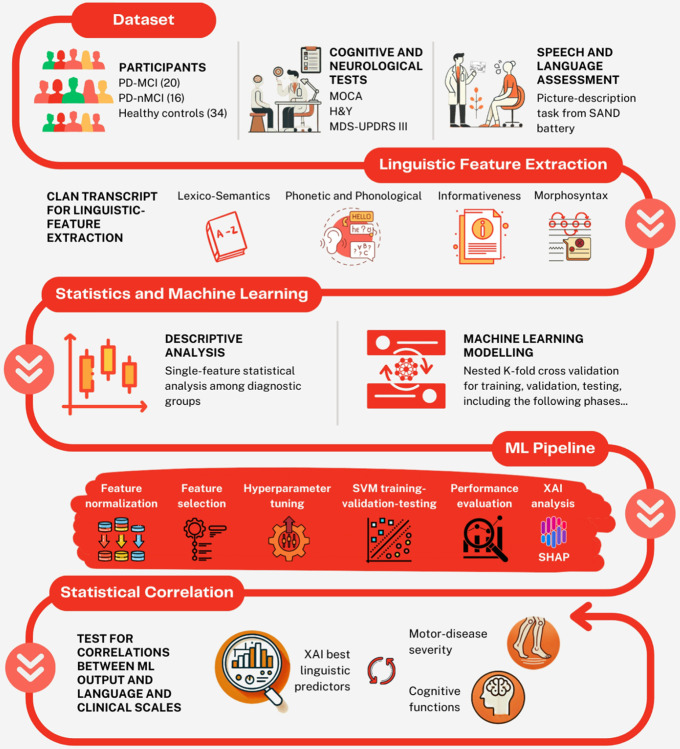
Analysis pipeline. : patient enrollment, and examinations; : computational linguistic analysis; : descriptive analysis of the different subgroups and classifications; : different phases in ML modeling; : association between linguistic optimal predictors and clinical scales.

Our present approach was conceived to tackle these gaps. We recruited individuals with PD alongside HCs, and asked them to describe a picture to elicit their verbal production. We investigated the use of semi-automatic linguistic analysis in the classification of PD by means of Artificial Intelligence (AI) to detect linguistic markers linked to the condition in an Italian sample, which remains under-represented in the PD literature. We then conducted four binary classifications: all patients with PD against HCs, PD-nMCI against HCs, PD-MCI against HCs, and PD-nMCI against PD-MCI. The pipeline is depicted in Fig. 1.

Variables of interest were extracted from participants’ connected speech samples using the Computational Linguistic ANalysis (CLAN) software (TalkBank, Helwan University^33^). Linguistic features were jointly analyzed and individually ranked reflecting the relative weights or contributions of the different linguistic domains within PD clinical profile. Here we focused on action verbs and morphosyntax to confirm previous findings and explored a larger number of complementary features that embrace several linguistic domains to help capture the array of different language impairments that can be seen in PD.

Based on previous findings, we hypothesized that individuals with PD would be robustly identified through linguistic features extracted from the connected speech, specifically by lexico-semantic features. Second, as discussed above^6^, we posited that action verbs would possess strong discriminatory power in the PD-nMCI subgroup since they are likely to be affected before broader cognitive disturbances arise due to damage to frontostriatal networks. Third, we anticipated that PD-MCI patients would be impaired in several linguistic categories and best discriminated by lexico-semantic patterns, mirroring the atrophy in frontostriatal networks. Lastly, we explored correlations between the top three optimal linguistic features, and clinical scales assessing motor symptom severity and global cognitive efficiency. We predict that a specific impairment in action verbs in PD-nMCI would be associated with motor severity as established via the MDS-UPDRS-III, supporting the claim of interaction between motor skills and specific disturbances of action semantics. Moreover, we predict that general cognitive impairment would be proportional to the presence of linguistic deficits in PD-MCI as general cognitive impairment is likely to impact executive functions involved in linguistic features. Testing these predictions aims to pave the way for objective, cost-effective, and sustainable approaches to scalable markers for PD.

## Results

The results of the descriptive analysis of demographic and clinical variables for all the binary classifications are outlined in Table S1 in the Supplementary Materials.

For the machine learning (ML) analysis, a total of thirty-five linguistic features were extracted from the connected speech. When samples were divided into training, validation, and test sets, patients with PD, patients with PD-nMCI, patients with PD-MCI, and HC sample sizes ensured more samples than the average number of features in each training iteration, as shown in Table S2 in Supplementary Materials.

### All patients with PD vs. all HCs classification

The results of the linguistic features’ descriptive analysis of all patients with PD vs. all HCs classification are outlined in Table S3 in the Supplementary Materials.

The Support Vector Machine (SVM) performance (%) are shown in Table 1. The classification performance yielded an accuracy of 77.14% ± 13.80 and an area under the curve (AUC) of 76.67 ± 13.35.

**Figure 2:**
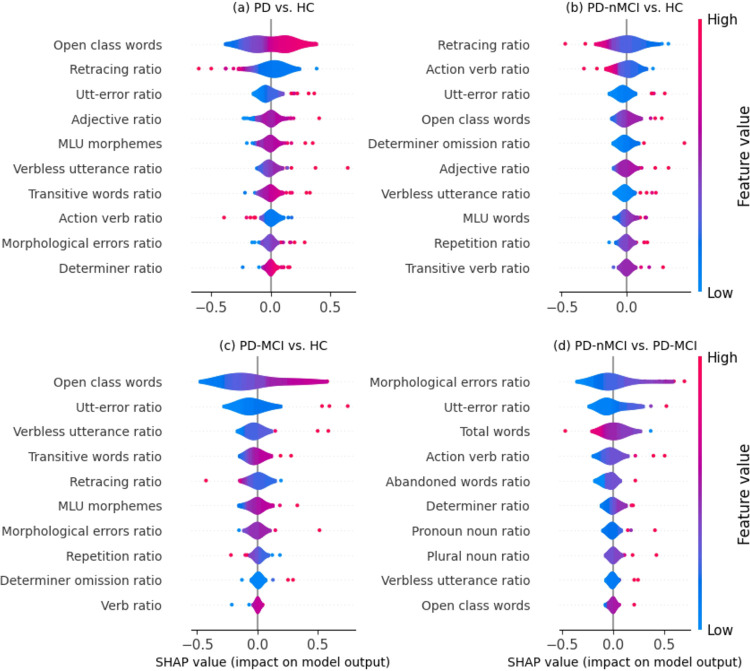
Absolute ranking and effect of linguistic features on model prediction, according to Shapley values, in the four classifications: a) all patients with PD against all HCs b) patients with PD-nMCI against HCs, c) patients with PD-MCI against HCs, and d) patients with PD-nMCI against patients with PD-MCI.

According to Shapley values, the absolute ranking and feature effects on the prediction are shown in Fig. 2a. The optimal linguistic features were open class words, retracing ratio, utterance-error ratio (utt-error ratio), adjective ratio, and mean length utterance (MLU) in morphemes. In particular, more open class words, utt-error ratio, adjectives ratio, MLU calculated in morphemes, and fewer retracings ratio increased the likelihood of being classified as PD.

In Table 2, the partial correlation coefficients between the top three optimal linguistic features, i.e., open class words, retracing ratio, utt-error ratio, and clinical scales, are presented. In the PD population, no significant association was found between the optimal linguistic features and the clinical scales.

**Figure 3:**
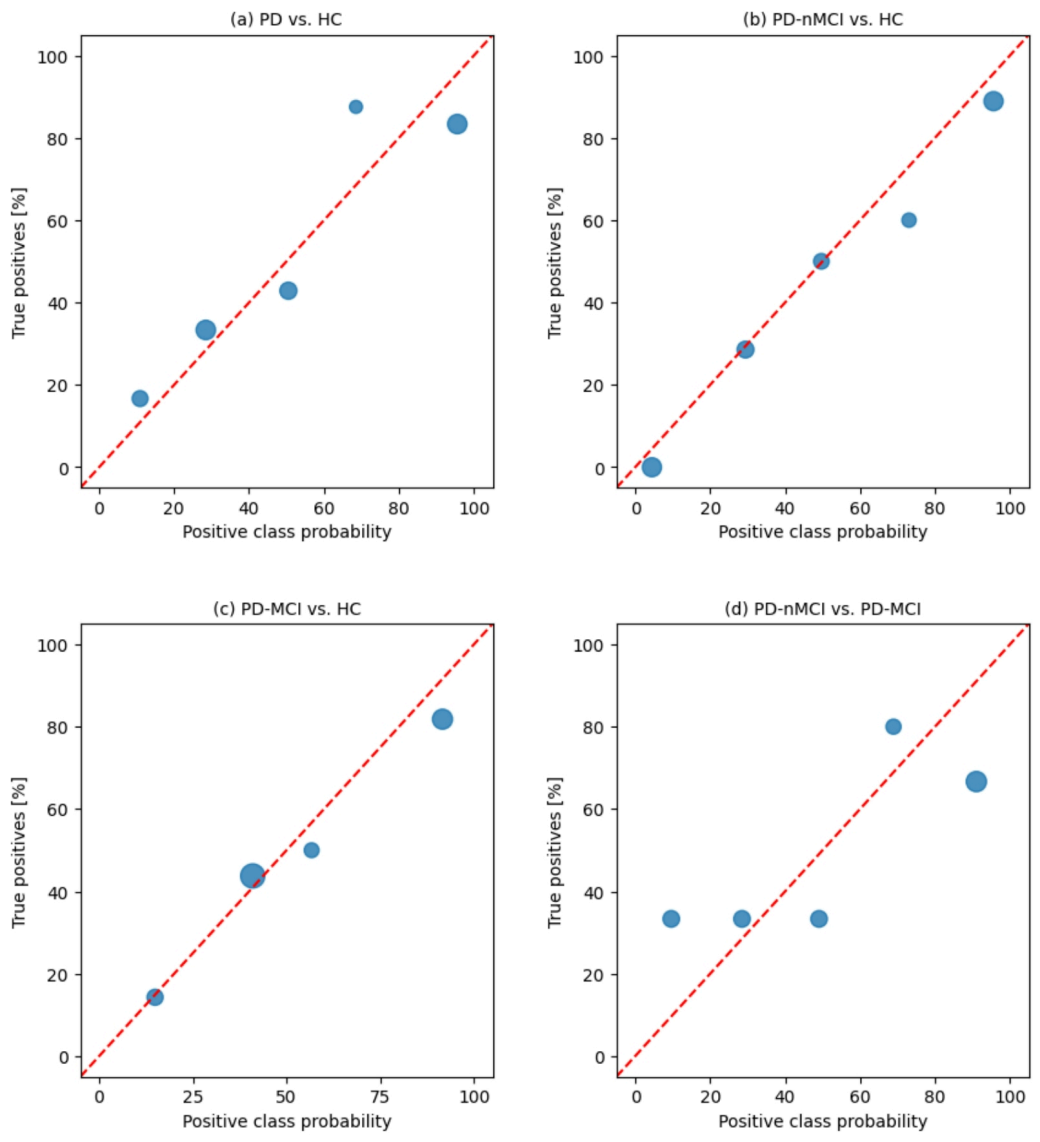
Calibration bubble chart showing the percentage of true positives in relation to the probability of belonging to the positive class. The red dashed line is the diagonal, representing the expected quality trend of all four binary classifications: a) all patients with PD and all HCs classifier, b) patients with PD-nMCI and HCs, c) patients with PD-MCI and HCs, and d) patients with PD-nMCI and patients with PD-MCI.

Lastly, as shown in Fig. 3a, the SVM model is well-calibrated. In bins 0–20, 20–40, and 60–80, the SVM model results slightly underestimate the risk of disease, whereas in bins 40–60 and 80–100, the risk of disease is slightly overestimated.

### Patients with PD-nMCI vs. HCs classification

The results of the linguistic features’ descriptive analysis of patients with PD-nMCI vs. HCs classification are outlined in Table S4 in Supplementary Materials.

The classification performance (%) are shown in Table 1. Specifically, accuracy and AUC were equal to 84.17 ± 18.19, 85.00 ± 17.48, respectively.

According to Shapley values, the absolute ranking and feature effects on the prediction are shown in Fig. 2b. The optimal linguistic features were retracings ratio, action verb ratio, utt-error ratio, open class words, and determiners omission ratio. In particular, more utt-error ratio, open class words, determiner omission ratio, and fewer retracing ratio and action verb ratio increase the likelihood of being classified as PD-nMCI.

In Table 2, the partial correlation coefficients between the top three optimal linguistic features, i.e., retracing ratio, action verb ratio, utt-error ratio, and clinical scales, are presented. In the PD-nMCI population, the action verb ratio was significantly inversely related to the MDS-Unified Parkinson’s Disease Rating Scale (MDS-UPDRS III) (P = 0.05 and *ρ* = −0.50).

Finally, as shown in Fig. 3b, the SVM model is well calibrated. In bins 20–40, and 40–60, the SVM model perfectly estimates the risk of disease, whereas in the remaining bins, the risk of disease is slightly overestimated.

### Patients with PD-MCI vs. HCs classification

The results of the linguistic features’ descriptive analysis of patients with PD-MCI vs. HCs classification are outlined in Table S5 in the Supplementary Materials.

The classification performance (%), as shown in Table 1, yielded an accuracy and an AUC both equal to 82.50 ± 16.87.

According to Shapley values, the absolute ranking and feature effects on the prediction are shown in Fig. 2c. The optimal linguistic features were open class words, utt-error ratio, verbless utterance ratio, transitive words ratio, and retracings ratio. In particular, more open class words, utt-error ratio, verbless utterance ratio, transitive words ratio, and fewer retracing ratio increase the likelihood of being classified as PD-MCI.

In Table 2, the partial correlation coefficients between the top three optimal linguistic features, i.e., open class words, utt-error ratio, verbless utterance ratio, and clinical scales, are presented. In the PD-MCI population, the verbless utterance ratio was significantly inversely related to the Montreal Cognitive Assessment (MoCA) (P < 0.01 and *ρ* = −0.60).

Lastly, as shown in Fig. 3c, the SVM model is well-calibrated. In bins 0–25, and 25–50 the SVM model perfectly estimates the risk of disease, whereas in the remaining bins, the risk of disease is slightly overestimated.

### Patients with PD-nMCI vs. PD-MCI classification

The results of the linguistic features’ descriptive analysis of patients with PD-nMCI vs. PD-MCI classification are outlined in Table S6 in the Supplementary Materials.

The SVM performances (%), shown in Table 1, specifically, accuracy and AUC were, respectively, equal to 75.83 ± 23.72, 75.00 ± 26.35.

According to Shapley values, the absolute ranking and feature effects on the prediction are shown in Fig. 2d. The optimal linguistic features were morphological error ratio, utt-error ratio, total words, action verb ratio, and abandoned words ratio. In particular, more morphological errors ratio, utt-error ratio, action verb ratio, abandoned words ratio, and fewer words produced increase the likelihood of being classified as PD-MCI.

As shown in Fig. 3d, the SVM model is not well calibrated. In bins 0–20, 20–40, and 60–80, the SVM model underestimates the risk of disease, whereas in the remaining bins the risk of disease is overestimated.

**Figure 4:**
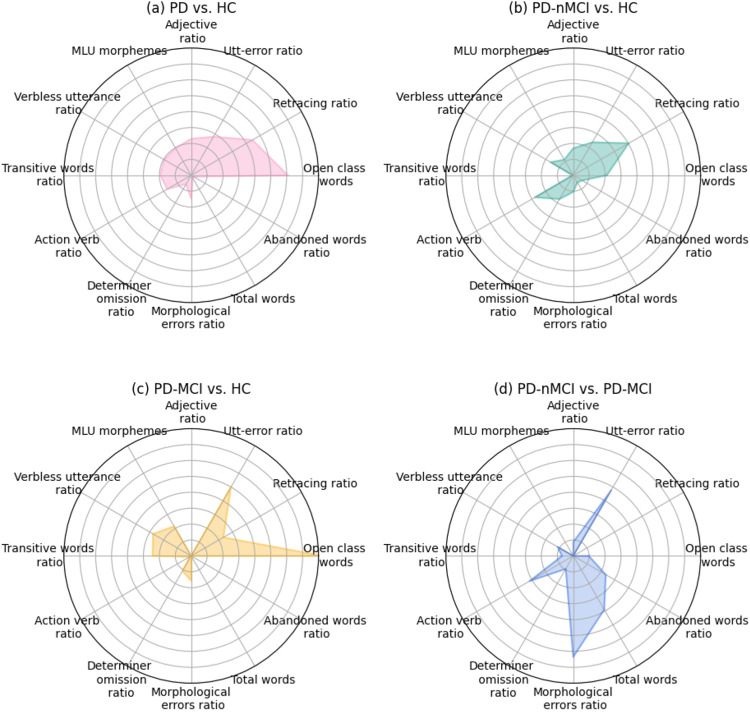
Radar plot of the absolute contribution of the optimal linguistic features, according to Shapley values, in each binary classification: a) all patients with PD against all HCs b) patients with PD-nMCI against HCs, c) patients with PD-MCI against HCs, and d) patients with PD-nMCI against patients with PD-MCI.

The comparison of the absolute contribution of the overall optimal linguistic features, according to Shapley values, in all four binary classifications is shown in Fig. 4

## Discussion

This work aimed to quantify the production of part of speech in patients with PD presenting with and without MCI and to develop a language-tailored approach to determine whether and which linguistic markers can classify patients into PD phenotypes automatically. We implemented a computational linguistic analysis combined with an explainable ML pipeline to establish a signature of PD (relative to HCs) based on measures of natural language. We also looked at the differences between PD-nMCI and PD-MCI to deepen our understanding of the role of cognitive impairment on the linguistic profile of PD. Assorted linguistic measures combined with ML algorithms can lead to a successful semiautomatic classification of individuals with PD, even in all subgroups. In all the classifications performed, lexico-semantic and morphosyntactic features were the most discriminatory. Furthermore, significant associations were found between lexico-semantic and morphosyntactic features with, respectively, MDS-UPDRSIII and MoCA. In a previous systematic review, we have shown that linguistic measures can serve as reliable biomarkers for monitoring disease progression in PD with encouraging classification performance; however, the evidence on the applications of machine learning algorithms to classify speech and language patterns resulted to be still scant^25^.

In this study, classification between all patients and HCs employing multivariate linguistic markers reached good performance metrics (i.e., AUC = 77%, accuracy = 77%). While previous ML studies on PD have reported good classification results; our model accuracy was higher than those reported up to date (i.e.,^6,29–31^). Conceivably, this is because of our multivariate approach, which integrates a higher variety of linguistic information than the more standard unidimensional approaches in previous works that use information derived from one linguistic domain (i.e.,^6,10,23^). Our results also demonstrated strong performance in distinguishing different PD phenotypes, achieving an AUC/accuracy of 85/84% for PD-nMCI vs. HCs, 83/83% for PD-MCI vs. HCs, and 75/76% for PD-nMCI vs. PD-MCI, while also providing a well-calibrated probability output, as indicated by the calibration plots. This underscores the model’s ability to handle subtle differences in linguistic profiles, a critical advantage given the heterogeneity of PD-related language impairments. Our findings indicate that, even without dementia, individuals with PD experience difficulties in processing different aspects of language.

Delving into feature importance, we found that overall patients vs. HCs were best discriminated by Phonetic and Phonological, Lexico-semantic, and Morphosyntactic features. At the Phonological level, our results revealed significant differences in the retracing ratio (e.g., reformulations of a message), suggesting that patients with PD may present a different behavior in verbal monitoring. Collectively, these and previous findings documented difficulties in PD in detecting speech errors compared to HCs, suggesting a dysfunction of the frontal system, which is known to be implicated in self-monitoring^34,35^. A difficulty in recognizing and self-correcting speech errors may serve as a possible indicator of attentional and self-monitoring deficits in PD.

At the Lexico-semantic level, our findings support earlier research showing that in PD a notable decline is observed in the generation of conceptual units, a reduction in informative content as expressed by open class words^36^. Open class words in Italian are nouns, verbs, adjectives, and some adverbs, while articles, pronouns, prepositions, and conjunctions are closed classes. While open class words convey most of the semantic meaning of the sentence, closed class words are heads introducing syntactic sentences. Only two studies^37,38^ analyzed the production of open class words in PD, observing a similar pattern. The author interpreted the use of fewer open class words related to possible compensatory strategies that patients adopt to convey in fewer open class words as much information about a concept as possible. Nevertheless, it remains to be explored whether open class words convey informational units consistent with the picture description task. While this aspect was not investigated in our study, future research could examine the correlation between the number of open class words produced and the number of informational units conveyed to better understand whether they align.

At the morpho-syntactic level, individuals with PD produced more errors in utterances and a greater MLU in morphemes. The first describes errors involving larger segments of an utterance or the whole utterance, including agrammatic and paragrammatic utterances, jargon (meaningless speech), empty speech, perseverations, and circumlocutions; the latter refers to the total number of phonologically well-formed morphemes divided by the number of utterances produced. Our findings concord with the existing literature that has consistently documented a reduction at the message level and morpho-syntactic simplification in the spoken language of patients with PD, which may become more apparent if patients are cognitively compromised^24,39,40^. This pattern leads to a reduction of information contents and mirrors the deficits observed in the idea formulations seen in PD^20^, and it has usually been associated with working memory and processing speed^41^.

The explainability analysis revealed that a fewer number of retracing ratio (Phonetic and Phonological level), action verb ratios, and a greater number of open class words (at the Lexico-semantic level), utterance error ratios, and determiner omission ratio (at the Morphosyntactic level) increase the possibility of being classified as PD-nMCI. Retracing ratio was the most important linguistic predictor, and as discussed above, this pattern may be attributed to a reduced ability to self-monitor speech errors. Action appraisal deficits refer to verbal expressions processed by motor and premotor areas whose meaning is related to bodily movement and have been already identified as a hallmark for distinguishing PD-nMCI from HCs, even in a preclinical stage of the disease^42^. Remarkably, this variable was found to supersede traditional cognitive assessments in sensitivity^17^. It has been proposed as a marker of motor network atrophy due to the dysfunction of frontobasal connections and altered dopamine levels leading to difficulties in using action verbs. A greater number of determiner omission ratio (Morphosyntactic level) in PD suggests that patients may struggle with closed class words, which are syntactically crucial to structuring a sentence, leading to lower synatctic complexity^39^.

When looking at the classification between PD-MCI vs. HCs, we found that patients differentiated from HCs across multiple linguistic domains: retracing ratio (at the Phonetic and Phonological level); open class words (at the Lexico-semantic level); utt-error ratio, verbless utterance ratio, transitive words ratio (at the Morphosyntactic level). Interestingly, we found that PD-MCI demonstrates a lower number of retracing and produces a greater number of open class words as shown in PD-nMCI, suggesting that these deficits are unlikely to be caused by cognitive impairment alone, but rather they may be language-tailored to the profile of PD. Concerning the latter three linguistic features we found that grammatical and syntactic processing is simplified in PD-MCI as they tend to use less verbs in sentences and the few reported are mainly transitive. In Italian, transitive verbs require two arguments (i.e., requiring a subject and an object). This may imply two important considerations: (i) grammatical language production appears to be associated with cognitive impairment, and more specifically, executive function deficits (that typify PD) may play a crucial role in the use of verbs^43^; (ii) the more the cognitive decline, the higher the use of transitive verbs (e.g., accusative verbs) that can represent a compensatory strategy in PD. By relying on verbs with more definite syntactic structure, like transitive verbs, patients with PD-MCI can organize their speech more effectively compared to the more open-ended intransitive structures. This might also be due to the greater predictability or clearer syntactic structure involved in transitive verbs, which may be easier for PD patients to process or access cognitively than the more flexible structure of intransitive verbs. However, this assumption needs to be better explored in the literature, as unfortunately previous studies did not analyze the proportion of transitive verbs produced by patients with PD; therefore, there are no data with which to compare the current findings.

Different relevant targets in differential diagnoses between PD-nMCI vs. PD-MCI were found in the present study; higher number of abandoned words ratio (Phonetic and Phonological level), action verb ratio (Lexico-semantic level), morphological errors ratio, utterance error ratio (at the Morphosyntactic level) and decreased total words (Discouse level) increased the probability of being classified as PD-MCI. Abandoned words refer to phonological fragments which can lead to hesitations, slower speech, or pauses. This aspect of speech may cause the patient to abandon words mid-sentence, stemming from challenges in the initial stages of language production, such as conceptualization and formulation, as well as difficulties with motor planning and articulation^39^. Action-concept has been found to discriminate between PD-nMCI and PD-MCI, suggesting its potential to distinguish different phenotypes of PD. Bocanegra and colleagues [2017]^21^ demonstrated a selective deficit for high-motion action verbs in PD-nMCI, attributing this impairment to the disruption of motor network integrity. Our findings corroborate this result, as action verbs were among the critical features distinguishing PD-nMCI from HCs. However, our study expands on this by employing an automated, feature-rich linguistic analysis and identifying additional variables, such as retracing ratio and utterance-error ratio, which contribute to classification performance. Another paper by García et al. [2022]^6^ demonstrated that action-related semantic metrics are particularly sensitive in differentiating PD subgroups from controls. While our approach did not focus exclusively on action-laden narratives, the inclusion of a diverse array of linguistic features, such as morphological errors and utterance structure, enabled high classification performance across multiple comparisons. Notably, our results suggest that while action-related deficits are central, broader morphosyntactic impairments also play a significant role, especially in discriminating the two phenotypes. Finally, patients with PD-MCI tended to produce less verbal output and utterances that were shorter, less syntactically complex, and less informative than patients without cognitive involvement. Deficiencies in terms of informational content and morphosynactic level also have been documented in previous studies of spoken language in PD and appear to be related to cognitive changes^44^.

The relationship between linguistic markers and clinical scales reveals a significant negative partial correlation between the ratio of action verbs and the severity of motor symptoms (MDS-UPDRS III) in PD-nMCI. This finding indicates that action semantics is linked to the extent of motor impairment, supporting our hypothesis that action concepts are disrupted specifically in PD-nMCI. Also, the verbless utterance ratio was significantly inversely related to global cognitive efficiency (i.e., MoCA) in PD-MCI, suggesting that having a cognitive deficit may impact the integrity of language mechanisms at the morphosyntactic level.

A notable strength of the present study is the explainability of our ML pipeline, achieved through the retracing ratio, action verb ratio, and utterance error ratio, our approach enhances interpretability, which is vital for clinical applications. The ability to identify key linguistic markers tied to underlying cognitive and motor impairments not only facilitates accurate classification but also offers potential for individualized assessments and targeted interventions. Digital linguistic markers have significant potential to improve clinical practices in the management of PD, offering benefits in early detection, monitoring, personalized interventions, and enhancing patient engagement in their own care. Early detection of a language-tailored pattern in PD could support clinical decision-making and intervention, helping to improve communication as well as quality of life for patients. The integration of automated classification approaches into standard clinical workflows may lead to better outcomes for patients, as they can facilitate the evaluation of the effectiveness of speech and language therapies or medications.

There are emerging applications and software designed for patients to record their speech and their language for analysis. Recently, a new device called the Toolkit to Examine Lifelike Language (TELL) has been developed to capture linguistic markers of neurodegenerative disorders through automated speech analysis^45^. These applications can offer real-time feedback and help in therapy as they can support remote monitoring of patients through telehealth platforms. Home-based management and interventions in neurodegenerative disorders reflect a growing recognition of the need for personalized care that enhances patient autonomy, improves quality of life, and facilitates access to ongoing support in a familiar environment^46^.

Compared to literature, our multimodal approach allowed the identification of additional linguistic markers, reinforcing the claim that such features are grounded in motor networks. Our ML approach lies in its ability to integrate a diverse set of linguistic features, enabling nuanced and robust classification of PD subgroups and HCs. The use of the RFE technique ensured the selection of the most informative linguistic markers while minimizing overfitting, contributing to high classification performance across classifications.

Additionally, our model’s nested CV design, coupled with hyperparameter optimization, ensures the generalizability of the results by reducing the risk of data leakage and overestimation of performance metrics, thus strengthening the reliability and reproducibility of our findings.

Despite its strengths, this study has some limitations to consider. First, the sample size, though comparable to or higher than previous studies on the same topic, is relatively modest. This limitation is particularly relevant for subgroup analyses, such as PD-nMCI versus PD-MCI, where small sample sizes may reduce the statistical power and generalizability of the findings. Future studies should aim to replicate these results in larger, more diverse cohorts. Second, while our approach utilized a comprehensive linguistic feature set, the lack of neuroimaging data (e.g. Magnetic Resonance Image-MRI or Positron Emission Tomography-PET) limits the ability to correlate linguistic impairments directly with structural or functional brain changes. Incorporating such imaging modalities in future studies could provide a more detailed understanding of the neural mechanisms underlying the observed linguistic deficits. Third, all language samples were collected during the “ON” phase of dopaminergic medication. While this provides a more stringent test of linguistic impairments, it also introduces variability that may not fully capture the impact of medication states on linguistic performance. Future work should consider evaluating participants in both “ON” and “OFF” medication phases to assess the potential modulatory effects of dopamine on language production.

To the best of our knowledge, this study is the first attempt to perform an AI-driven linguistic analysis on Italian-speaking subjects to identify PD and distinguish between subgroups. While this allowed us to address a significant gap in the literature, linguistic and cultural differences may limit the generalizability of our findings to other languages and populations. In the future, cross-linguistic studies are needed to determine whether the identified linguistic markers and classification models are universally applicable or language-specific^47,40^.

In conclusion, our study builds on and extends the insights from these foundational works by leveraging computational linguistic analysis and ML to characterize PD-related language profiles. While previous studies have focused on specific linguistic domains or task-specific metrics, our approach integrates a comprehensive feature set and robust classification models, providing a detailed characterization of linguistic impairments in PD and related MCI subgroups. These findings reinforce the potential of automated language analysis as a scalable and clinically relevant tool for early diagnosis and phenotyping of neurodegenerative diseases. Finally, clinicians should bring their attention to language-specific aspects that might not be considered in PD and should be prepared to address adequately the communicative needs of patients with PD.

## Methods

### Participants

Patients with a clinical diagnosis of idiopathic PD according to the current criteria of the UK Parkinson’s Disease Society Brain Bank^1^ were consecutively enrolled in the study. The inclusion criteria were (1) availability of an audiotaped language examination to allow an offline analysis of connected speech, (2) MoCA score ≥ 17 following the Italian-specific cut-off^48,49^, (3) Italian native speaker, (4) sufficiently intelligible speech such that the intended target could be determined for the majority of words, and (5) intact or corrected auditory and visual functions. Patients were excluded in the case of (1) major psychiatric disorders^50^, (2) organic illness affecting the brain according to the International Classification of Diseases^51^, (3) significant history of head injury, (4) deep brain stimulation antecedents, (5) history of drug or alcohol addiction; and (6) history of linguistic deficits before PD symptoms onset, based on neurological examination.

We prospectively and consecutively enrolled 80 participants, including 46 Italian-speaking patients with PD patients and 34 matched HCs, recruited at the Laboratory of Neuropsychology of the Istituti Clinici Scientifici Maugeri IRCCS of Bari, Italy, between February 2023 and July 2024. Of the 46 patients consecutively enrolled in the study, 10 out of 46 patients were excluded based on the second inclusion criterion, leaving a total of 36 cases, who met all inclusion criteria.

Subsequently, patients with PD were further subdivided into two subgroups according to the presence of Mild Cognitive Impairment (MCI), following the level II criteria of the Movement Disorder Society Task Force^52^: n = 16 PD-nMCI, and n = 20 PD-MCI. The administered neuropsychological test battery is described in the Supplementary Materials.

A group of HCs was enrolled from a convenience sample of volunteers recruited in the same hospital. HCs underwent a multidimensional assessment, including neurologic and neuropsychological evaluation, and were included only if the results were in the normal range. PD-nMCI and PD-MCI groups were matched to healthy controls (HCs, n = 20) by age, gender, and education, with 12 HCs shared between subgroups. For direct comparison, 16 PD-nMCI patients were selected and matched to 16 PD-MCI cases.

Patients were tested during the “ON” phase of the anti-parkinsonian medication, which was converted to Levodopa equivalent daily dose (LEDD) using a previously published formula^53^. Disease severity was established using the Hoehn and Yahr Scale (H&Y)^54^. Non-motor and motor symptoms and their complications were examined using MDS-UPDRS III^55^.

All subjects provided their informed written consent to participate in the study and to perform the comprehensive clinical, speech and language, and neuropsychological evaluations. The study protocol was approved by the Institutional Review Board of the IRCCS Giovanni Paolo II Hospital (No. Prot. 1195 approved on 27th February 2023). All the procedures were performed according to the Declaration of Helsinki.

### Oral picture description

We elicited connected speech by the picture description task from the Screening for Aphasia in NeuroDegeneration battery (SAND)^56,57^. Participants were seated at a desk in a quiet room, positioned in front of a microphone (Shure Beta 87A) connected to an amplifier (Scarlett 2i2 3rd Generation, Focusrite) that in turn was connected to a computer. Patients were instructed as follows: *“Take a look at this picture, tell me what you see, and try to talk in sentences.”*. If the participant paused during the first minute of production, the examiner encouraged them to add more details to their observations using complete sentences. The audio files were recorded and saved in .wav format anonymously using Audacity (https://www.audacityteam.org/). The latter was used to preprocess the audio track by removing the intervention of the examiner. The recordings were transcribed using Whisper (OpenAI)^58^, an automatic speech recognition encoder-decoder Transformer-based model, which retrieves transcripts in .txt format files. Transcripts were manually reviewed during the production of CLAN ones in CHAT format (Codes for the Human Analysis of Transcripts), which is a specialized standard transcription format used in language research for analyzing spoken and written language data. CLAN software analyzes transcripts by combining transcription and several codes that are used by transcribers to recognize, analyze, and take note of phenomena in transcribed speech.

### Connected speech measures derived using CLAN

Language analysis was performed through the use of CLAN^59^, following three steps: (i) transcript coding in CHAT format; (ii) POS tagging; and (iii) feature extraction. Transcription in CHAT format was conducted by two independent transcribers. For reliability purposes, discrepancies between the two transcribers were solved by consensus criteria for utterance segmentation established by Saffran et al., 1989^60^.

In addition, CLAN holds programs for morphosyntactic analysis to perform complete part-of-speech (POS) tagging for every word present in the transcript. CLAN’s MOR library was employed as a POS tagger to identify nouns, verbs, adjectives, pronouns, adverbs, prepositions, and conjunctions. Determiners and action verbs were manually identified.

Thirty-five linguistic features, defined in Table S7 in the Supplementary Materials, were clustered according to four linguistic levels defined in previous literature^25^:

1. Informativeness, including total words and idea density;
2. Phonetic and Phonological, including phonological errors ratio, repetition ratio, retracing ratio, and abandoned words ratio;
3. Lexico-semantics, including open class words, verb ratio, noun ratio, plural noun ratio, adjective ratio, adverb ratio, closed class words, determiner ratio, pronoun ratio, preposition ratio, noun verb ratio, pronoun noun ratio, semantic (SEM) errors ratio, and action verb ratio;
4. Morphosyntactic, including total utterances, MLU in words, MLU in morphemes, transitive verb ratio, intransitive verb ratio, transitive words ratio, intransitive words ratio, determiner noun ratio, preposition-utterance ratio, verbless utterance ratio, utterance-error ratio, morphological errors ratio, determiner omission ratio, preposition omission ratio, and preposition substitution ratio.

All these features were used in machine learning analysis.

### Statistical analysis

The above-mentioned features were statistically compared between groups: (a) all patients with PD vs. all HCs, (b) PD-nMCI vs. HCs, (c) PD-MCI vs. HCs, and (d) PD-nMCI vs. PD-MCI.

Demographical, clinical, and linguistic features of participants are presented using descriptive summary statistics. Normal distributions of quantitative variables were tested using the Shapiro-Wilk test. Thus, continuous normal distributed variables were reported as mean ± standard deviation (M ± SD), continuous non-normal distributed variables were reported as median and interquartile range (Median (IQR)), and frequency and percentages (%) for all categorical variables.

A statistical approach based on the null hypothesis significance two-sided test was used to test differences between groups, using the parametric t-test for normal distributed variables or the non-parametric U-Mann Whitney test for non-normal distributed variables. Magnitude differences between continuous variables were assessed using Wilcoxon’s effect size (ES) and 95% confidence interval (CI). False Discovery Rate (FDR) correction^61^ was used to adjust p-values for linguistic features in multiple comparisons. Differences between categorical variables were assessed using the Chi-squared test.

To identify any possible partial correlation, adjusted for potential confounders (i.e., age, sex, education, LEDD, and disease duration), between linguistic and clinical variables, the parametric Pearson’s or nonparametric Spearman’s correlation coefficients according to variable distribution were calculated. The significance level adopted was 5% (P ≤ 0.05), with 95% CI. Data were analyzed using RStudio (version 2024.04.2).

### Feature Selection, Hyperparameter tuning, and Machine learning analysis

Linguistic features were used to classify participants in each group: (a) all PD vs. all HCs, (b) PD-nMCI vs. HCs, (c) PD-MCI vs. HCs, and (d) PD-nMCI vs. PD-MCI. A classifier for each group was implemented. The ML model employed in each group comparison was the SVM^62^, which has proven useful in the same classification task using similar features^6,26^.

Recursive Feature Elimination (RFE)^63^ was used to perform feature selection. RFE is a wrapper-type feature selection algorithm, using a ML model as the core of the method. RFE starts by training the core model with all the features in the training set, after performing the importance ranking of all features, it discards the least important features and retrains the model until it defines the subset of features that maximizes performance, i.e. the selected features. The core model used was the Logistic Regression, the minimum number of features to be selected was set to five, and the scoring parameter to ‘roc-auc’.

The SVM hyperparameters, i.e., the regularization factor (C), the kernel function (kernel), and the radius of the area of interest in the case of the gaussian kernel (gamma), were optimized using a grid search strategy^64^, in which a set of values is defined for each hyperparameter, as shown in Table S8 in the Supplementary Materials and combined to form a grid of hyperparameters. The best set of hyperparameters is defined by searching over the aforementioned grid to maximize the performance of SVM on the validation set. The scoring parameter used was the ‘roc-auc’. The best model obtained from hyperparameter tuning was used as ML model to perform the classification task.

ML models were trained using a 10-fold Nested-Cross-Validation approach. The original dataset was split into 10 subsets: 9 out of 10 were used in an inner training-and-validation loop to perform feature selection and hyperparameter tuning; the remaining subset was then used in the outer loop as a test set for the performance evaluation of the SVM. This procedure was repeated 10 times until all subsets were used as a test set in the outer loop.

In the inner loop, the training-and-validation set, obtained from the outer loop, was further split into 5 subsets: 4 out of 5 were used as training set, whereas the remaining one as validation set. Training, validation, and test data were normalized using standard normalization. Normalization was fit on the training data and then applied to validation and test data.

In each iteration, the selected features and the optimal hyperparameters were estimated in the inner loop as those able to maximize the AUC of the classification, whereas model performance was computed on the test set of the outer loop (see Table S6 in the Supplementary Materials for details on the size of the training, validation, and testing sets).

Accuracy, sensitivity, specificity, AUC, PPV, NPV, and F1-score were computed to evaluate the SVM-model performances across all 10 folds, and presented as Mean ± SD.

In addition, we evaluated the calibration of the SVM model by dividing the entire dataset used for testing into N bins, each bin representing a probability interval of belonging to the positive class. The number of bins for each comparison was selected according to the dataset dimensionality (i.e., all PD vs all HCs: 5 bins, PD-nMCI vs HCs: 5 bins, PD-MCI vs HCs: 4 bins, and PD-nMCI vs PD-MCI: 5 bins). For each bin, we calculated the probability (averaged across folds of the nested cv) of each sample to belong to the positive class and the average number of true positives in that bin. It is worth noting that the single-subject probability for each fold is given by the output probability of belonging to the positive class estimated by the SVM classifier. The model calibration was represented using a bubble chart having on the x-axis the probability of the positive class (%) and on the y-axis the percentage of true positives. Each bubble has a marker size proportional to the number of samples belonging to each bin. Data were analyzed using the scikit-learn library in Python (v3.11).

### Explainability analysis

Model explainability was carried out through the use of SHapley’s Additive exPlanation (SHAP). SHAP is a post-hoc model agnostic method that employs a cooperative game theory notion known as Shapley values, which dates back to 1950^65^. The Shapley values define the features’ importance, i.e., the contribution of each feature in the model prediction process. According to Lundberg and Lee^66^, to calculate the effect of each feature, it is necessary to retrain the model *f* on a subset of features *S* ⊆ *F*, where *F* is the total number of features. Therefore, to compute the effect of *x*_*i*_, representing the feature of the *i-th* sample, a model *f*_*S*∪ *i*_ is trained using *x*_*i*_, whereas a model *f*_*S*_ is trained without it, obtaining respectively *f*_*S*∪ *i*_ (*x*_*S*∪ *i*_) and *f*_*S*_ (*x*_*S*_) predictions. These are compared, as follows:

$$f_{S\cup i}\left(x_{S\cup i}\right)-f_{S}\left(x_{S}\right)$$

where *x*_*S*_ represents the values of the input features in the set. The aforementioned difference is calculated for all potential subsets because the impact of omitting a feature depends on other features *S* ⊆ *F* \ *i* in the model.

The Shapley values are the weighted average of all potential variations:

$$\varphi_{i}=\sum_{S\subseteq F\setminus \left\{i\right\}}\frac{\left|S\right|!(\left|F\right|-\left|S\right|-1)!}{\left|F\right|!}\left[f_{S\cup \left\{i\right\}}\left(x_{S\cup \left\{i\right\}}\right)-f_{S}\left(x_{s}\right)\right]$$

where *|F|!* represents the amount of feature value permutations placed before the *j* − *th* feature, while *(|S|−|F|−1)!* represents the number of feature value permutations after the *j* − *th* feature value, and *|S|!* is the total amount of permutations. The value of *φ*_*i*_ is the Shapley value of the single *x*_*i*_ feature. SHAP values must be calculated for each feature combination, which takes exponential time.

The SHAP strength lies in its ability to provide predictive explainability values both at the global level, i.e., over the entire population under examination, but also at the local level, i.e., over the individual subject in the population.

In each iteration, after model training and testing, the SHAP explainer was trained using the training set, and Shapley values were calculated using the test set. In each iteration, the features selected by RFE are different, thus the Shapley values of the unselected features are set equal to zero. The Shapley values of each test set were placed within a matrix of size (n_samples)-by-(total number of features), used to compute the absolute ranking and effect of each feature on the prediction. Data were analyzed using the shap library (v0.45.1) in Python (v3.11).

## Figures and Tables

**Table 1:** SVM model performances (%) in all PD vs. HCs, PD-nMCI vs. HCs, PD-MCI vs. HCs, and PD-nMCI vs. PD-MCI

All PD vs. all HCs						
Accuracy	Sensitivity	Specificity	AUC	PPV	NPV	F1-score
77.14 ± 13.80	72.50 ± 20.80	80.83 ± 22.92	76.67 ± 13.35	85.67 ± 16.01	74.33 ± 17.27	75.79 ± 16.13
PD-nMCI vs. HCs						
Accuracy	Sensitivity	Specificity	AUC	PPV	NPV	F1-score
84.17 ± 18.19	80.25 ± 25.82	90.00 ± 21.08	85.00 ± 17.48	90.00 ± 21.08	85.00 ± 19.95	81.67 ± 19.95
PD-MCI vs. HCs						
Accuracy	Sensitivity	Specificity	AUC	PPV	NPV	F1-score
82.50 ± 16.87	75.00 ± 35.36	90.00 ± 21.08	82.50 ± 16.87	92.59 ± 14.70	85.00 ± 19.95	84.44 ± 15.63
PD-nMCI vs. PD-MCI						
Accuracy	Sensitivity	Specificity	AUC	PPV	NPV	F1-score
75.83 ± 23.72	75.00 ± 35.36	75.00 ± 35.36	75.00 ± 26.35	75.93 ± 34.47	83.33 ± 25.00	86.67 ± 15.12

**Table 2:** Partial correlations between linguistic features and clinical scales.

PD			
	Open class words	Retracing ratio	Utt-error ratio
**MoCA**	−0.17^a^	−0.04^a^	−0.28^b^
	*0.33*	*0.81*	*0.09*
**MDS-UPDRS III**	−0.24^a^	0.21^a^	0.04^a^
	*0.16*	*0.22*	*0.81*
PD-nMCI			
	Retracing ratio	Action verb ratio	Utt-error ratio
**MoCA**	−0.16^b^	0.33^b^	−0.36^a^
	0.54	0.22	0.18
**MDS-UPDRS III**	−0.26^a^	−0.50^a^	−0.12^a^
	*0.32*	**0.05**	*0.66*
PD-MCI			
	Open class words	Utt-error ratio	Verbless utterance ratio
**MoCA**	−0.06^a^	−0.39^a^	−0.60^a^
	*0.79*	*0.09*	<0.01
**MDS-UPDRS III**	−0.35^a^	0.04^b^	0.10^b^
	*0.13*	*0.87*	*0.68*

a Spearman’s correlation,

b Pearson’s correlation

## Data Availability

The dataset generated and analyzed during the current study is available from the corresponding author.
